# Prevalence of Non-Engineered Buildings and Population at Risk for a Probable Earthquake: A Cross-Sectional Study from an Informal Settlement in Tehran, Iran

**Published:** 2020-01

**Authors:** Soraya FATHOLLAHI, Sahar SAEEDI MOGHADDAM, Mohammad Ali MANSOURNIA, Mahmoud RAHIMI, Mehdi ZARE, Ali ARDALAN, Ali SHEIDAEI, Niloofar PEYKARI, Shohreh NADERIMAGHAM, Farshad FARZADFAR

**Affiliations:** 1 Department of Emergencies and Disaster Health, School of Public Health, Tehran University of Medical Sciences, Tehran, Iran; 2 Non-Communicable Diseases Research Center, Endocrinology and Metabolism Population Sciences Institute, Tehran University of Medical Sciences, Tehran, Iran; 3 Endocrinology and Metabolism Research Center, Endocrinology and Metabolism Clinical Sciences Institute, Tehran University of Medical Sciences, Tehran, Iran; 4 Department of Epidemiology and Biostatistics, School of Public Health, Tehran University of Medical Sciences, Tehran, Iran; 5 Department of Urban Planning, Faculty of Architecture and Urban Planning, Islamic Azad University, Central Tehran Branch, Tehran, Iran; 6 International Institute of Earthquake Engineering and Seismology, Tehran, Iran; 7 Harvard Humanitarian Initiative, Harvard University, Cambridge, USA; 8 Department of Biostatistics, School of Paramedical Sciences, Shahid Beheshti University of Medical Sciences, Tehran, Iran; 9 Development of Research and Technology Center, Deputy of Research and Technology, Ministry of Health and Medical Education, Tehran, Iran

**Keywords:** Earthquake, Non-engineered buildings, Population, Informal settlements, Disasters

## Abstract

**Background:**

Constructions in informal settlements not respected any applying rules, regulations of urban planning, and building codes with high population density, are the municipality challenge. We aimed to identify level of buildings seismic vulnerability and population at risk in Tehran’s Farahzad informal settlement in 2017.

**Methods:**

In this observational cross-sectional study, residential buildings were assessed for seismic performance of constructions. We screened 160 buildings according to Iranian national guidelines by Rapid Seismic Visual Screening Method as a tool to calculate and determine Level of Retrofitting (L_R_) scores of buildings. We also interviewed residents of the buildings to collect data regarding socio-demographic data, individual disability status, Disaster Assessment of Readiness and Training (DART) regarding household disaster preparedness, and time occupancy in the buildings.

**Results:**

Overall, 160 buildings with 209 households and 957 individuals were surveyed. 97.5% of buildings were formed of heavy construction materials. None of them were categorized as engineered buildings and L_R_ of residential buildings ranged from 82.4% to 163.8% with a mean 117.9%. L_R_ scores of more than 100% were capped as 100%. Vulnerable groups of the sample population include under-five years old (8.7%), 60 yr old and above (6.7%), and 9.1% of households had at least one disabled member. 16.7% of households were living in homes with dense area. The DART score for 94.3% of surveyed households was zero.

**Conclusion:**

Disaster managers in Tehran municipality must design and implement a comprehensive risk reduction plan in poor urban areas as vulnerable regions for earthquake hazard.

## Introduction

The unpredictable nature and destructive impact of earthquakes is the public health concern in less developed countries. During the last decade's buildings collapse due to earthquakes cause the vast impact and casualties in the world (1). More consolidated efforts are needed to achieve Sustainable Development Goals (SDGs) related to safe housings in disasters (2). Iran is one of the top ten countries with the highest number of earthquakes occurrence and more casualties. In recent decades the most lethal earthquakes have occurred in Iran and more than 180,000 people were died (3–6). Geographical location of Tehran is located in high seismicity region. Although this megacity has historical experience of earthquakes in the past but not have had any earthquakes for 150 yr (7–9). A third of the urban population in developing world yet live in informal settlements: this proportion for western Asia is 25% in 2014 (10, 11). Increasing population growth in Iran and migration from rural areas has led to creation of informal settlements, located in areas adjacent to most active faults. Urban planning rules and building codes are mostly ignored in constructing such settlements and create significant challenge to the municipality (12–14).

Recognizing key concept such as addressing built environment risks is necessary for better understanding disasters consequences in local, regional, and national levels (15–17). In pre-disaster phase for geological hazards, attention must be focused on seismic performance of existing buildings because people are not killed by earth shaking but are killed by structural collapse (18–21). According to Sendai Framework for Disaster Risk Reduction “The first priority is risk understanding”. Proactive approach in mitigation phase must be developed and designing epidemiological researches for risk identifying should be integrated with other disciplines (17, 22, 23).

Recognizing the built environment risk for geological hazards is crucial (5, 10). Densely populated, informal settlements in Tehran have been developed without any urban planning and infrastructures (24). In these settings, pre-earthquake-impact survey and identification of exposure real population is needed to more accurate data of life-losses (10, 12, 22, 23). Hence, identifying unsafe residential buildings is essential in earthquake-prone area. Determination of why and how deaths may be caused by potential earthquakes is essential for planning and preparing in future events (3, 25, 26).

The epidemiological investigations in disasters field could be integrated with other disciplines like civil engineering and seismology in seismic events and useful to improve rationalize decision making among public health disaster managers (22).

The study aimed to identify level of buildings seismic vulnerability, population at risk and their readiness for disasters in Tehran’s Farahzad neighborhood.

## Materials & Methods

### Design

**Fig. 1:**
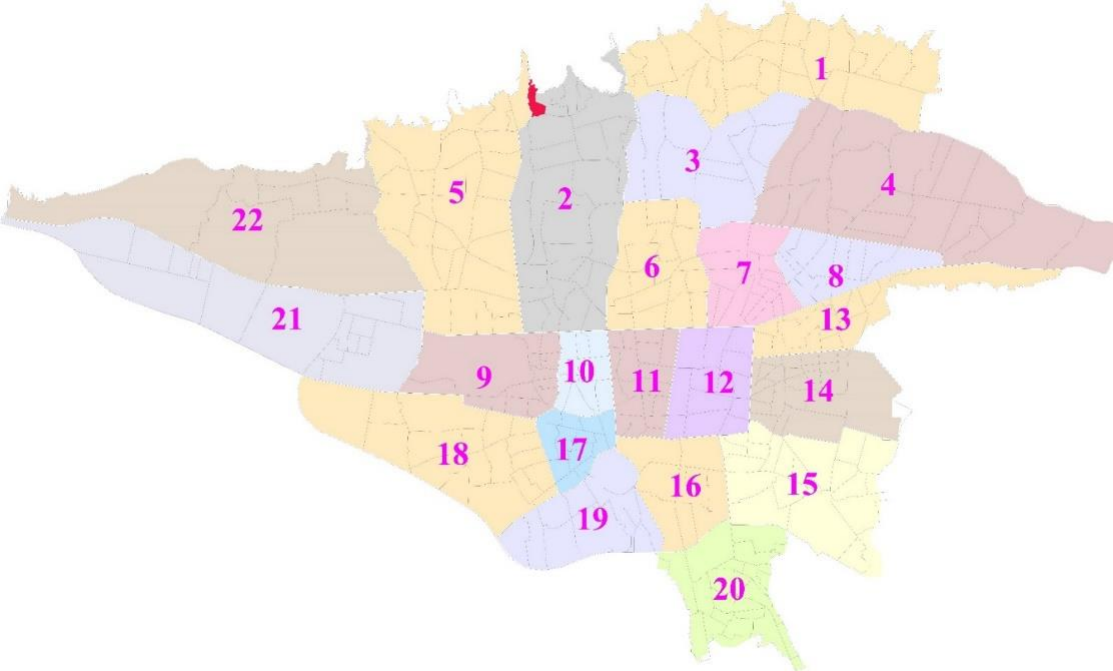
Farahzad neighborhood location on 22 urban regions of Tehran

This research is a cross-sectional study on the seismic performance of residential buildings in Farahzad informal settlements colored in red in Fig. 1. The most probable earthquake scenarios were determined for Tehran with parameters such as Modified Mercalli Intensity (MMI) Scale and Peak Ground Acceleration (PGA) in consulting with seismologist in International Institute of Earthquake Engineering and Seismology (IIEES) of Iran. Two scenarios considered were classified as best and worst cases given by MMI: VII, PGA: 0.25 and MMI: IX, PGA: 0.45 respectively. Residential buildings assessing were conducted in collaboration with a civil engineer.

### The survey tools

The study instrument, a questionnaire based on broader designed study objectives, consisted of three sections. First section consisted of building assessment form, prepared according to the Iranian national guidelines for Rapid Visual Seismic Screening (RVS.No376). Rapid Visual Screening of Buildings for Potential Seismic is a method that requiring limited engineering analysis based on information from visual observations in field and on-site measurements (27). The data was later used to calculate level of vulnerability (L_R_) scores. L_R_ is percentage of building vulnerability. It is used for prioritizing buildings in term of seismic retrofitting. Average L_R_ is calculated from 10 items include: land slope (L_1_), soil type (L_2_), foundation (L_3_), structural wall (L_4_), type of roof (L_5_), protrusion (L_6_), building plan (L_7_), openings (L_8_), number of stories (L_9_), and building quality (L_10_). The formula for calculating L_R_ is given by,

$$\begin{array}{c} \begin{array}{c} L_{R}=0.45\times [L_{3}+L_{4}+L_{5}+L_{6}+L_{7}]\times L_{1}\\ \times L_{2}\times L_{8}\times L_{9}\times L_{10}\\ \times (7.5A-1) \end{array} \end{array}$$

Where *A* denotes Peak Ground Acceleration (PGA) based on the building codes in Iran. The cutoff point for including these building for detailed assessment was score less than 75%.

More information on the structural system, construction period, building size, floor area per person, and occupancy type were obtained and they considered as first section of questionnaire. We also took photographs of sampled buildings to use in assessing the vulnerability.

Moreover, we collected socio-demographic data, individual disability, instantaneous occupancy in buildings was considered as second section of questionnaire. The third section of questionnaire consist of disasters household’s preparedness and observed from Disaster Assessment of Readiness and Training (DART) questionnaire. DART program is in accordance with Disaster Risk Reduction Office Programs in Iranian Ministry of Health and Medical Education (MOHME).

### Sampling

Sample size was calculated based on the odds ratio for building destruction of the latest epidemiological study regarding building collapse in Iran earthquakes (12). Overall, 160 buildings were determined with systematic randomized sampling. Before commencing the field survey, information on the structural system, age, or occupancy not existed in Tehran municipality. Therefore, for solving these barriers a list of Farahzad’s land parcels was attained from Tehran Municipality ICT Organization (TMICTO) that it is characterized by eight numbers code. In addition, a map with approximately 1: 3500 mm magnitude was provided for accurate identifying samples in field. Inclusion criteria for selecting the land parcels to participate in the study was that residential building has been constructed in this land.

In addition, for the gathering high quality of data, training sessions were conducted for buildings screener. Data were collected using questionnaire-based interview by a team. The head of household was eligible for interview; however, any adult as a household member was eligible as a respondent. Every household in buildings was interviewed without any consideration of the number of building stories.

In line with ethical consideration pertain to conducting RVS method, taking photographs of constructions as well as collecting socio-demographic data of household written consent of respond person have been gotten. In addition, in order to appreciate households’ collaboration in this survey, the gifts were provided for households. The response rate was high (more than 98%) in buildings which households were not present after three times referral, we have to replace the nearest another building. Data analysis was performed using Stata version 12.

Ethical approval for the study was obtained from Tehran University of Medical Science (ID: IR.TUMS.VCR.1395.486) and the work was funded by Non-Communicable Diseases Research Center of Tehran University of Medical Sciences.

## Results

There were 209 households, which includes 957 individuals who were living in 160 buildings in Farahzad informal settlements in the survey in 2017.

**Fig. 2:**
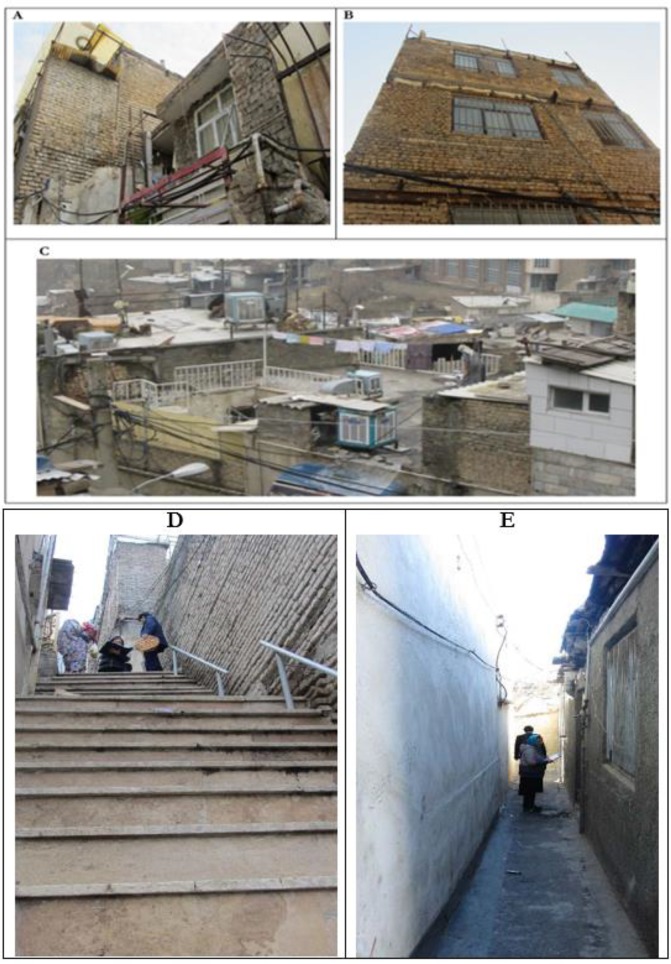
Sampled residential buildings in Farahzad neighborhood (2017 Jan). (a) Non-engineered buildings sample (b) Crowded situation of buildings (c) sampled constructions with heavy material (d) Stairway pathway (e) Narrow pathway

**Fig. 3:**
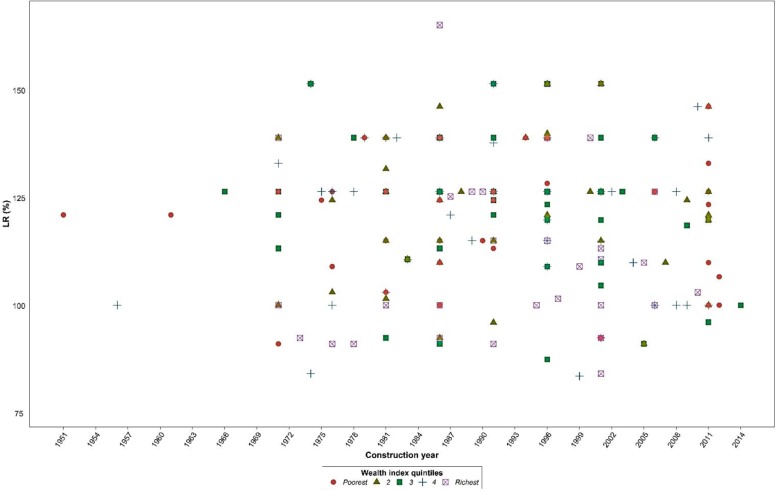
Distribution of buildings’ construction year by percent of L_R_ and Wealth index quintiles of families

About 50.9% of surveyed population was female and the average age was 28.6 yr (SD ±18.6). Vulnerable groups of the sample population include under-five years old, 60 yr old and above, and disable individuals were 8.7%, 6.7%, and 9.1% of families had in their households, respectively. The average household size was five persons (Table 1). None of the 160 buildings was categorized as engineered buildings (Fig. 2A). L_R_ of residential buildings in sampled ranged from 82.4% to 163.8% with a mean equal to 117.99%. There is no association between construction year and mean of L_R_ in Wealth index quintiles of families, derived by Principal Component Analysis (PCA) (Fig. 3). In situation with L_R_ more than 100% considered as 100%. Mean of L_R_ in the poorest and the richest quintiles of wealth index were 99.5 and 96.9, respectively. A Kruskal-Wallis H test showed that there was a statistically significant difference in L_R_ between the five wealth index quintiles (*P*-value=0.001).

**Table 1:** Characteristics of Individuals

***Variable***	***Category***	***Number***	***Percent***
Age (yr)	Under-5	83	8.7
	5–14	176	18.4
	15–59	634	66.2
	60 and more	64	6.7
Sex	Female	487	50.9
	Male	470	49.1
Years of schooling (for whom more than 6 yr)	Illiterate	175	20.5
	Primary	263	30.8
	Secondary	165	19.3
	High school	47	5.5
	Diploma	128	15.0
	Associate of science	20	2.3
	Bachelor of science	46	5.4
	Master of science	10	1.2
Job (for whom more than 15 yr old)	Employed	250	29.8
	Unemployed	81	9.7
	Income without employment	14	1.7
	Student	201	24.0
	Housekeeper	259	30.9
	Other	34	4.1
Marital (for whom more than 10 yr old)	Married	456	57.4
	Widow	25	3.1
	Divorced	16	2.0
	Single	257	32.4
	Missing	40	5.0
Type of insurance	Iran Health Insurance Urban	219	22.9
	Iran Health Insurance Rural	1	0.1
	Social Health Insurance	278	29.1
	Military Force Health Insurance	8	0.8
	Others	23	2.4
	Don't know	18	1.9
	Without insurance	406	42.4
	Missing	4	0.4
Complementary insurance (for whom with basic insurance)	Yes	41	7.4
	No	501	90.9
	Don’t know	5	0.9
	Missing	4	0.7
Time occupancy at 11:45	Yes	494	51.6
	No	463	48.4

Approximately, 97.5% of buildings are formed of heavy construction materials such as brick, concrete briquette, steel, and stone composition with sand-cement mortar and 2.5% was (clay, Adobe) with clay-mud mortar (Fig. 2B). Number of stories, 92.5% of buildings have 1 to 2 stories, and only 1.3% of building has four stories. The maximum and minimum percent of floor area per person in this survey respectively belonged to 7 to 10.9 m^2^ (29.7%), and 15 to 19.9 m^2^ (9.6%) (Table 1). Based on characteristics of 209 families 16.8% of households were lived in homes with 1 or 2 rooms (by considering kitchen and hall). Only 9.6% of households live in homes with 5 or more rooms (Fig. 2C). Farahzad’s dwellings have been constructed in narrow passageways with steep stepped streets (Fig. 2D, E), 61.3% of residential buildings have passageways between 1 to 3 m and 7.5% of these buildings are located in alleys with 10 to 12 m widths (Table 2). The average distance of these residential buildings to the nearest governmental emergency medical center was 4736 in m, maximum and minimum distance were 6800 ad 3200 in m, respectively. 61.3% of residential lands located in street with width of 1 to 3 m, while only 11.9% of them were located in street with 7 m and more.

**Table 2:** Characteristics of households

***Variable***	***Category***	***Number***	***Percent***
Households Nationality	Iranian	160	76.6
	Afghani	49	23.4
Ethnicity	Persians	51	31.9
	Kurds	43	26.9
	Lurs	17	10.6
	Azari	46	28.8
	Gilaks, Mazandaranis	2	1.3
	Turkmen	1	0.6
Disabled	Yes	19	9.1
	No	190	90.9

Variables related to household’s disaster preparedness status such as DART score for 94.3% of surveyed households were zero means that these population did not have any information regarding disaster preparedness and training.

## Discussion

In recent decades, rural texture of Farahzad as a leisure zone and river valley have changed and linked to Tehran municipality, immigrants from the other provinces have been informally located in this steep slope or hill sloped area (24). In poor urban area the chance to find cheaper land parcel and construct low-cost building provide opportunities for building contravention in informal settlements (28). The spatial typology of informal urban settlements is different from the planned urban settlements.

The data revealed all sampled dwellings have been constructed with heavy materials, assembled without any structural engineering design. According to L_R_ scores calculated using the Iranian RSV method, none of the buildings considered qualify for further considerations regarding retrofitting process.

Our analysis showed the buildings construction year and household’s socio-economic status do not influence their LR levels significantly. Overall, 61% of passageways in Farahzad were steep stepped streets and alleys less than 6.0 m wide. Furthermore, the DART scores has revealed the majority of the population do not have any disaster awareness and readiness plan. There were also no emergency medical centers in the area and the average distance to these centers was 4736 m.

The lack of infrastructures and services in Farahzad informal settlements such as quality of roads, passageways hinders search and rescue efforts that can exacerbate the impact of disasters and create challenges to emergency operations in earthquake events.

Inadequacies in construction standards and accessibility due to road closure coupled with the high population in this area. More vulnerable groups such as elderly people, under-five children and disabled persons in earthquake in overcrowded housings, could complicate search and rescue operations after an earthquake occurrence. Although earthquakes are relatively infrequent in an event, heavy damage is expected in Farahzad neighborhood.

The RVS approach has broadly conducted in US and other countries as a practical and simple instrument to prioritizing the buildings for seismic vulnerability considerations (29). A survey conducted (30) to evaluating buildings earthquake safety in mid-American communities, of the 295 buildings surveyed in Carbondale, 51% have scored less than or equal to 2.0. Although higher scores mean better seismic performance, ATC-21 mandates more investigation by a professional engineer experienced in seismic design (27, 30). In “La Milagrosa” informal settlement, detailed structural analyses are needed and confirmed all sampled buildings provide inadequate seismic performance (25). “FEMA 154” was applied for rapid visual evaluation of 1000 structures in two districts of Jeddah City (31). Only 46% of surveyed buildings in old neighborhood (Al-Balad) and 17% in other district needed minor restoration. Seismic performance of 71 school buildings constructed in Khuzestan Province was evaluated with Iranian RVS.No376 and FEMA154 guidelines. About 25% of these buildings had risk of excesses vulnerability and only 2% of them had risk of collapse. Remaining percentage had lower risk for seismic vulnerability (32). Aria method was applied for seismic vulnerability assessment of Qazvin city buildings that is a qualitative methods for assessing buildings vulnerability in earthquake. The majority of masonry buildings and some of steel and concrete buildings in Qazvin city were highly vulnerable in severe and moderate earthquakes (33).

According to these researches that aforementioned in process of seismic vulnerability screening, their occupants of buildings not be considered and surveyed. Additional work has been carried out in our study in order to generate accurate data and identifying population at risk of death. Whilst, globally a lot of modern casualty estimation models exist for earthquake (8, 22, 34, 35) and are based mainly on the vulnerability of the built environment, risk factors such as population characteristics, socioeconomic status, physical disability and population disasters preparedness (22, 36–38) not considered in these models. The present study in field of disaster epidemiology in addition screening seismic vulnerability performance of buildings have considered socio-demographic characteristics of their occupants.

Achieving first priority of the Sendai Framework on Disaster Risk Reduction (39) requires identifying the population at risk, vulnerable groups, poor socioeconomic status, poor urban infrastructures and unsafe buildings. The majority of epidemiological studies on identifying mortality risk factors in earthquakes are conducted in the aftermath of earthquake (5, 12, 40, 41). To reduce the burden of earthquake casualties due to non-predictable events, risk assessment in high seismic prone zones should be considered as first priority in mitigation phase of disaster (16, 18, 22).

Indeed poor quality dwellings that constructed with assembled material (brick and corrugated iron) have found in poor neighborhoods of urban centers and informal settlements (42) and Farahzad in line with these situations has extremely bad condition. Unfortunately, minimum careful spatial planning and urban design not existed in Farahzad neighborhood.

Institutional weakness is evident in Tehran municipality subjecting the area to increased unplanned settlements. In general, documents that contain permits, plans, and structural calculations have not been found for Farahzad settlements. Seismic behavior of these constructions emphasizes the need for developing adequate procedures for seismic strengthening of existing structures. Construction technical profile in seismic prone area and poor settings urban is more essential for disaster management cycle. The quality of road networks in Tehran’s informal settlements have largely remained unchanged. Urban planning should, therefore, be revised and modified in such areas. Metropolitan authorities must carry out mandatory regular monitoring of protective strip for raw land and tenure rules as well as constructing according to standard codes in this area. There is a need to communicate the results of these assessments to Farahzad neighborhood population to developing community-based earthquake preparedness programs. Despite the existing standard codes in Iran, all sampled buildings were unreinforced masonry buildings, assembled with heavy materials without any lateral loads design. Providing facilities for relocating densely populated unsafe areas must be implemented in urban poor settings regions.

Limitation: lack of neighborhood-based building inventories in term of numbers and building typology, postal codes and their occupants in Tehran municipality was limitation for determining samples. Therefore, we have to determine samples according to the list of Farahzad land parcels.

## Conclusion

Farahzad informal settlement buildings must be renewed and governmental supportive loans can be useful for building owners and motivated them to building reconstruction. Popular culture of earthquake preparedness is necessary for urban poor population. Disasters managers in Tehran municipality must have been a comprehensive risk reduction plan in poor urban area.

## Ethical considerations

Ethical issues (Including plagiarism, informed consent, misconduct, data fabrication and/or falsification, double publication and/or submission, redundancy, etc.) have been completely observed by the authors.
